# The Role of Indoxyl Sulfate in Exacerbating Colorectal Cancer During Chronic Kidney Disease Progression: Insights into the Akt/β-Catenin/c-Myc and AhR/c-Myc Pathways in HCT-116 Colorectal Cancer Cells

**DOI:** 10.3390/toxins17010017

**Published:** 2025-01-01

**Authors:** Yu Ichisaka, Chihiro Takei, Kazuma Naito, Manami Higa, Shozo Yano, Toshimitsu Niwa, Hidehisa Shimizu

**Affiliations:** 1 Graduate School of Natural Science and Technology, Shimane University, 1060 Nishikawatsu-cho, Matsue 690-8504, Shimane, Japan; 2 Faculty of Medicine, Shimane University, 89-1 Enya-cho, Izumo 693-8501, Shimane, Japan; 3 The Center for Integrated Kidney Research and Advance, Faculty of Medicine, Shimane University, 89-1 Enya-cho, Izumo 693-8501, Shimane, Japan; 4 Center for Community-Based Health-Care Research and Education (CoHRE), Head Office for Research and Academic Information, Shimane University, 223-8 Enya-cho, Izumo 693-8501, Shimane, Japan; 5 Shubun University, 6 Nikko-cho, Ichinomiya 491-0938, Aichi, Japan; 6 The United Graduate School of Agricultural Sciences, Tottori University, 4-101 Koyama-Minami, Tottori 680-8553, Tottori, Japan; 7 Faculty of Life and Environmental Sciences, Shimane University, 1060 Nishikawatsu-cho, Matsue 690-8504, Shimane, Japan; 8 Estuary Research Center, Shimane University, 1060 Nishikawatsu-cho, Matsue 690-8504, Shimane, Japan; 9 Interdisciplinary Center for Science Research, Shimane University, 1060 Nishikawatsu-cho, Matsue 690-8504, Shimane, Japan; 10 Institute of Agricultural and Life Sciences, Academic Assembly, Shimane University, 1060 Nishikawatsu-cho, Matsue 690-8504, Shimane, Japan

**Keywords:** Indoxyl sulfate, indole derivative, tryptophan metabolites, gut microbiota, chronic kidney disease, colorectal cancer, c-Myc, Akt, β-Catenin, AhR, EGFR

## Abstract

Epidemiological studies suggest an increased risk of colorectal cancer (CRC) aggravation in patients with chronic kidney disease (CKD). Our previous study demonstrated that indoxyl sulfate, a uremic toxin whose concentration increases with CKD progression, exacerbates CRC through activation of the AhR and Akt pathways. Consequently, indoxyl sulfate has been proposed to be a significant link between CKD progression and CRC aggravation. The present study aimed to investigate the roles of c-Myc and β-Catenin, which are hypothesized to be downstream factors of indoxyl sulfate-induced AhR and Akt activation, in CRC cell proliferation and EGF sensitivity in HCT-116 CRC cells. Indoxyl sulfate significantly induced CRC cell proliferation at concentrations exceeding 62.5 µM, a process suppressed by the c-Myc inhibitor 10058-F4. Indoxyl sulfate activated the Akt/β-Catenin/c-Myc pathway as evidenced by the Akt inhibitor MK2206, which decreased both β-Catenin and c-Myc protein levels, and the β-Catenin inhibitor XAV-939, which reduced c-Myc protein levels. The AhR antagonist CH223191 also inhibited c-Myc upregulation, indicating involvement of the AhR/c-Myc pathway. MK2206 partially attenuated the indoxyl sulfate-induced AhR transcriptional activity, suggesting that Akt activation influences the AhR/c-Myc pathway. MK2206, CH223191, and 10058-F4 suppressed the increase in EGFR protein levels induced by indoxyl sulfate, indicating that the Akt/β-Catenin/c-Myc and AhR/c-Myc pathways enhance the sensitivity of HCT-116 CRC cells to EGF. These findings indicate that the elevation of indoxyl sulfate levels in the blood, due to CKD progression, could worsen CRC by promoting the proliferation of CRC cells and enhancing EGF signaling. Therefore, indoxyl sulfate could potentially serve as a therapeutic target for CRC aggravation in patients with CKD.

## 1. Introduction

The kidneys, an essential organ in the urinary system, performs several critical functions. These functions include excretion of metabolites, regulation of water and electrolytes, maintenance of acid-base balance, and production of hormones, all of which aim to maintain homeostasis [1]. The Kidney Disease Improving Global Outcomes (KDIGO) guidelines define chronic kidney disease (CKD) as any structural or functional alteration in the kidneys that lasts more than 3 months [2,3]. After CKD is diagnosed, clinicians are advised to categorize patients into stages based on the estimated glomerular filtration rate (eGFR). The eGFR is calculated using the Chronic Kidney Disease Epidemiology Collaboration (CKD-EPI) formula. The CKD stages are defined as follows: Stage I (>90 mL/min/1.73 m^2^), Stage II (60–89 mL/min/1.73 m^2^), Stage IIIa (45–59 mL/min/1.73 m^2^), Stage IIIb (30–44 mL/min/1.73 m^2^), Stage IV (15–29 mL/min/1.73 m^2^), and Stage V (<15 mL/min/1.73 m^2^) [2,3]. CKD can profoundly affect health, possibly leading to irreversible damage to both the structure and function of the kidneys. Importantly, most individuals with renal dysfunction are at an elevated risk for cardiovascular disease, even without the need for dialysis or kidney transplantation [4]. Moreover, recent studies have clearly shown that CKD increases the risk of aggravating colorectal cancer (CRC) [5,6,7,8,9]. Additionally, a study has shown that patients with CRC and CKD have higher postoperative mortality rates and shorter median survival times than those without CKD [10]. These findings indicate that CKD can aggravate the prognosis of patients with CRC.

The oncogene c-Myc plays a critical role in regulating essential cellular processes, such as cell survival, apoptosis, differentiation, and proliferation. It also promotes angiogenesis, metastasis, and immune evasion [11,12,13,14,15,16]. Additionally, c-Myc plays a significant role in altering metabolism during CRC development and progression [12,16,17,18,19]. Moreover, patients with metastatic CRC (mCRC), especially those with RAS-BRAF wild-type genes who also show high levels of c-Myc expression, have significantly shorter periods of disease-free and overall survival than those with low levels of c-Myc [20]. β-Catenin regulates c-Myc expression and works together with TCF/LEF to start this process [21,22]. It also plays a role in promoting the growth, invasion, and metastasis of CRC cells [22,23,24]. Additionally, patients with CRC who present with nuclear β-Catenin and active PI3K/Akt pathway markers are at higher risk of metastasis. This association suggests potential interactions between these molecules [25]. AhR regulates c-Myc expression as well [26,27]. However, removal of AhR from colon epithelial cells may accelerate cell proliferation and exacerbate tumor growth in CRC models [28,29,30]. Conversely, studies have shown that activating the AhR pathway with TCDD enhances CRC cell survival and migration [31]. Moreover, the TDO2-AhR pathway is essential for facilitating liver metastasis in CRC [32]. AhR also influences resistance to chemotherapy [27,33,34]. Furthermore, inhibiting the Kyn-AhR axis could bolster anticancer immunity and potentially prevent colitis-associated cancers [35]. Thus, the complex role of AhR in CRC, marked by its interactions with specific ligands, could account for the observed diversity and occasional contradictions in the research findings.

*Fusobacterium nucleatum* (*F. nucleatum*) is linked to the worsening of CRC progression. This link is supported by the high presence of bacteria in the intestines during the advanced stages of the disease [36]. An increased intestinal abundance of *F. nucleatum* has also been reported in rat models of CKD [37]. *F. nucleatum*, a widely recognized bacterium, is known for its indole production [38]. Indole produced by intestinal bacteria such as *F. nucleatum* is transported from the colon to the liver via the portal vein. The liver metabolizes indole to indoxyl sulfate, a known uremic toxin [39]. Oral administration of *F. nucleatum* to rats with CKD led to higher serum indoxyl sulfate levels, thereby worsening CKD progression [37]. In patients with end-stage renal disease (ESRD), serum indoxyl sulfate levels begin at approximately 250 μM and may soar to approximately 550 μM [40]. Colon tissues are also exposed to various compounds in the bloodstream, with indoxyl sulfate accumulation observed in the colon tissues of rats with CKD [41]. Moreover, research has shown that indoxyl sulfate and related indole derivatives such as indole-3-acetic acid and skatole affect CRC cell function, proliferation, and inflammatory responses [42,43,44,45,46]. Consequently, indoxyl sulfate may contribute to the aggravation of CRC associated with CKD [47], and our study demonstrated that indoxyl sulfate may be implicated in the aggravation of CRC [48].

A study at the University of Tokyo, Japan (January 2001–December 2010) found that 21.8% of patients with primary CRC stages 0 to III who underwent curative resection were also diagnosed with CKD stages III to V [49]. Furthermore, individuals with early-stage CKD (stages I and II) showed blood indoxyl sulfate levels comparable to those of healthy individuals. However, in CKD stages III, IV, and V, these levels were significantly higher than those in the healthy group [50]. Additionally, elevated serum indoxyl sulfate levels have been observed in familial and sporadic CRC models in *Apc*^min/+^ mice [51]. Taken together, these findings indicate that aggravation of CRC may occur from CKD stage III onward. Notably, the significant increase in blood indoxyl sulfate levels in CKD stage III suggests a strong correlation with CRC aggravation at this stage. Indeed, findings from our previous study demonstrate that indoxyl sulfate induces the proliferation of CRC cells and enhances EGF sensitivity by upregulating EGFR expression [48]. However, the pathway downstream of AhR and Akt, which is activated by indoxyl sulfate, remains unclear. The present study focused on c-Myc and β-Catenin, which are assumed to be downstream of indoxyl sulfate-induced AhR and Akt activation, and aimed to analyze how these molecules affect the proliferation of CRC cells and enhance EGF sensitivity through the upregulation of EGFR protein levels in HCT-116 CRC cells.

## 2. Results

### 2.1. Indoxyl Sulfate Induces Proliferation of HCT-116 CRC Cells

**Figure 1:**
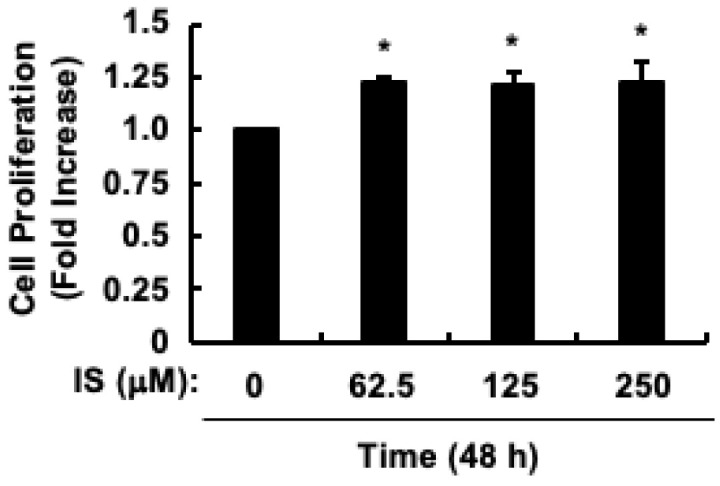
Impact of skatole on proliferation of HCT-116 CRC cells. Quantification of cell proliferation. The results are presented as the mean values ± SE of quadruplicates from three independent experiments. * *p* < 0.05, vs. 0 h. IS, indoxyl sulfate.

Exposing HCT-116 CRC cells to 250 μM indoxyl sulfate, which is the average serum concentration in patients with ESRD [40], for 24 h, led to observed cell proliferation [48]. However, since the effect of indoxyl sulfate stimulation for more than 24 h on the proliferation of HCT-116 CRC cells has not been confirmed, HCT-116 CRC cells were exposed to indoxyl sulfate at a maximum concentration of 250 µM for a longer period of 48 h to confirm the effect of long-term exposure to indoxyl sulfate. Stimulation of HCT-116 CRC cells with indoxyl sulfate for 48 h resulted in significant proliferation at concentrations above 62.5 µM (Figure 1). Therefore, we used 250 µM indoxyl sulfate in the present study, consistent with our previous research [48], which we assumed would be easier to analyze.

### 2.2. Indoxyl Sulfate-Induced Proliferation of HCT-116 CRC Cells Is Mediated by Increased c-Myc Expression

**Figure 2:**
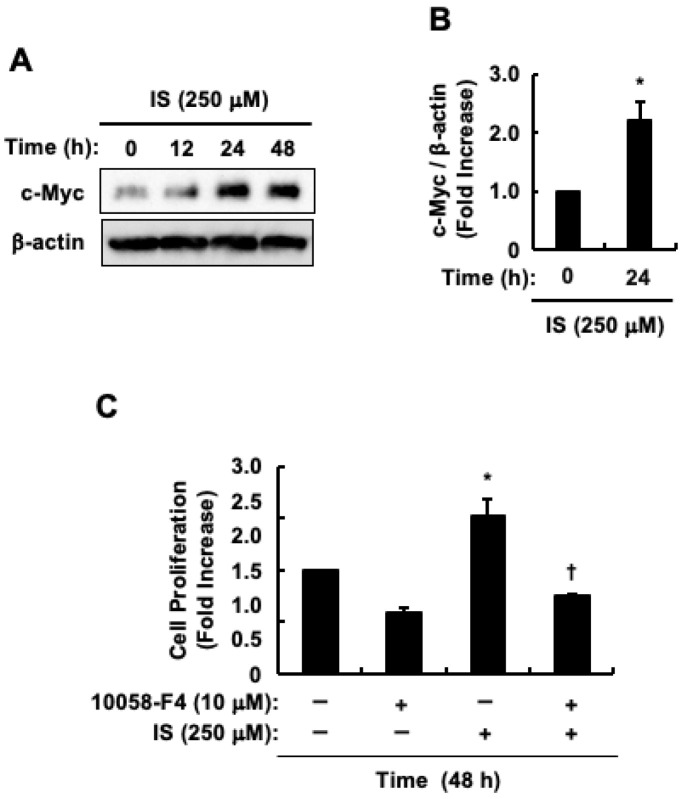
Impact of indoxyl sulfate on c-Myc protein levels and subsequent proliferation of HCT-116 CRC cells. (**A**) The effects of indoxyl sulfate on c-Myc and β-actin protein levels. (**B**) The band intensity of c-Myc in response to indoxyl sulfate. The band intensity of c-Myc was normalized to that of β-actin. The results are presented as mean values ± SE from three independent experiments. * *p* < 0.05, vs. 0 h. (**C**) The effect of 10058-F4 on indoxyl sulfate-induced cell proliferation. HCT-116 CRC cells were incubated with (+) or without (−) control (DMSO), indoxyl sulfate, or 10058-F4. The results are presented as the mean values ± SE of quadruplicates from three independent experiments. * *p* < 0.05, vs. control solution (DMSO). ^†^ *p* < 0.05, vs. indoxyl sulfate alone. IS, indoxyl sulfate.

We examined whether indoxyl sulfate was involved in the increase in c-Myc protein levels in HCT-116 CRC cells. c-Myc protein levels peaked 24 h after indoxyl sulfate stimulation (Figure 2A). Additionally, c-Myc protein levels significantly increased 24 h post-indoxyl sulfate stimulation (Figure 2B). Based on these results, we stimulated HCT-116 CRC cells with indoxyl sulfate for 24 h when examining c-Myc protein levels in subsequent analyses. The involvement of c-Myc in indoxyl sulfate-induced proliferation of HCT-116 CRC cells was examined using the c-Myc inhibitor, 10058-F4. Figure 2C shows that 10058-F4 significantly suppressed indoxyl sulfate-induced cell proliferation. Therefore, the increase in c-Myc protein levels may be implicated in the proliferation of HCT-116 CRC cells induced by indoxyl sulfate.

### 2.3. Indoxyl Sulfate Activates Akt/β-Catenin/c-Myc Signaling Pathway in HCT-116 CRC Cells

**Figure 3:**
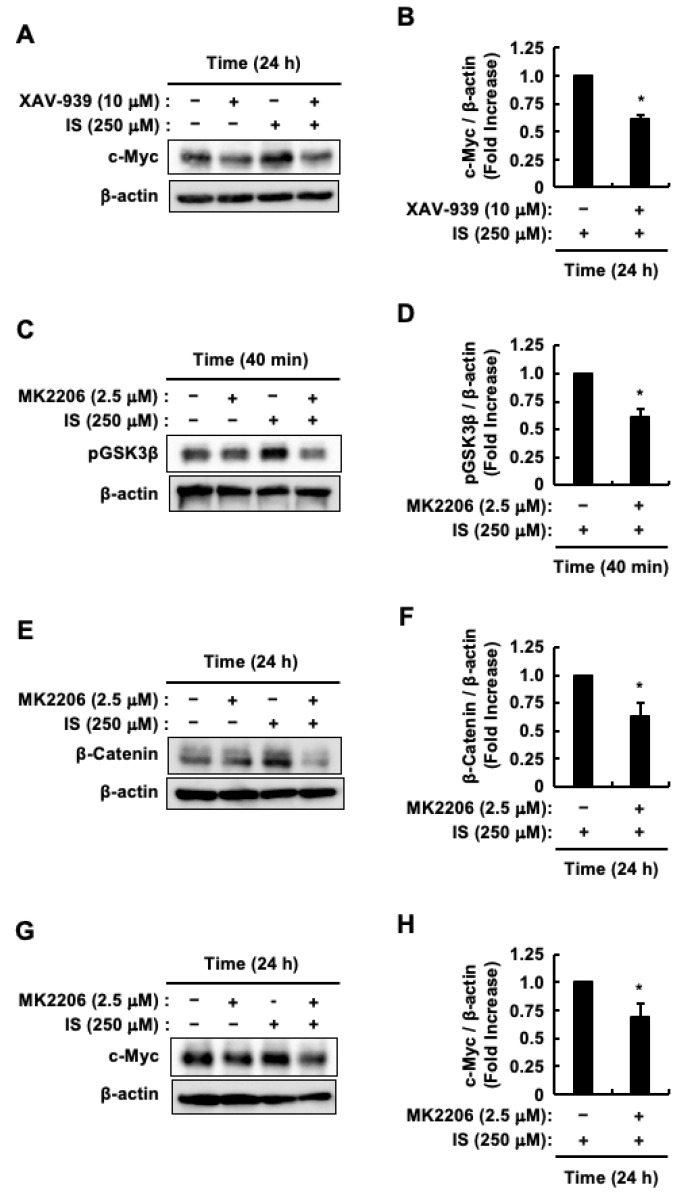
Impact of indoxyl sulfate on Akt, β-Catenin, and c-Myc signaling in HCT-116 CRC cells. HCT-116 CRC cells were incubated with (+) or without (−) control (DMSO), indoxyl sulfate, XAV-939, or MK2206. (**A**) The effects of XAV-939 on indoxyl sulfate-induced c-Myc and β-actin protein levels. (**B**) The band intensity of c-Myc in response to indoxyl sulfate alone and in combination with XAV-939. (**C**) The effects of MK2206 on indoxyl sulfate-induced phospho-GSK3β and β-actin protein levels. (**D**) The band intensity of phospho-GSK3β in response to indoxyl sulfate alone and in combination with MK2206. (**E**) The effects of MK2206 on indoxyl sulfate-induced β-Catenin and β-actin protein levels. (**F**) The band intensity of β-Catenin in response to indoxyl sulfate alone and in combination with MK2206. (**G**) The effects of MK2206 on indoxyl sulfate-induced c-Myc and β-actin protein levels. (**H**) The band intensity of c-Myc in response to indoxyl sulfate alone and in combination with MK2206. The band intensities of β-Catenin, phospho-GSK3β, and c-Myc were normalized to that of β-actin. The results are presented as mean values ± SE from three independent experiments for for (**B**,**D**,**F**,**H**). * *p* < 0.05, vs. indoxyl sulfate alone. IS, indoxyl sulfate; pGSK3β, phospho-GSK3β.

Considering β-Catenin’s regulatory role in c-Myc expression [21,22], we investigated its influence on the indoxyl sulfate-induced elevation of c-Myc protein levels, using the β-Catenin inhibitor XAV-939. XAV-939 suppressed indoxyl sulfate-induced increase in c-Myc protein levels (Figure 3A,B). Glycogen Synthase Kinase 3β (GSK-3β), which degrades β-Catenin [52], is inactivated by Akt through phosphorylation [53,54]. This process positions GSK-3β as a crucial mediator in the Akt/β-Catenin pathway within CRC cells, such as HCT-116 CRC cells [53,54]. Our previous study demonstrated that Akt is activated in HCT-116 CRC cells following 40 min of indoxyl sulfate stimulation [48]. Therefore, to confirm the phosphorylation of GSK-3β, a known substrate of Akt, HCT-116 CRC cells were pretreated with the Akt inhibitor MK2206 and then stimulated with indoxyl sulfate for 40 min. Figure 3C,D shows that MK2206 inhibits indoxyl sulfate-induced phosphorylation of GSK-3β. Since c-Myc expression increased following stimulation of HCT-116 CRC cells with indoxyl sulfate for 24 h, we subsequently analyzed its upstream molecule, β-Catenin, at the same stimulation time point. MK2206 decreased the increase in β-Catenin protein levels caused by indoxyl sulfate (Figure 3E,F). Moreover, MK2206 suppressed the increase in c-Myc protein levels induced by indoxyl sulfate (Figure 3G,H). Taken together, these results suggest that indoxyl sulfate-induced proliferation of HCT-116 CRC cells involves activation of the Akt/β-Catenin/c-Myc signaling pathway.

### 2.4. AhR Activation Induced by Indoxyl Sulfate Increases the Protein Levels of c-Myc But Decreases the Protein Levels of β-Catenin in HCT-116 CRC Cells

**Figure 4:**
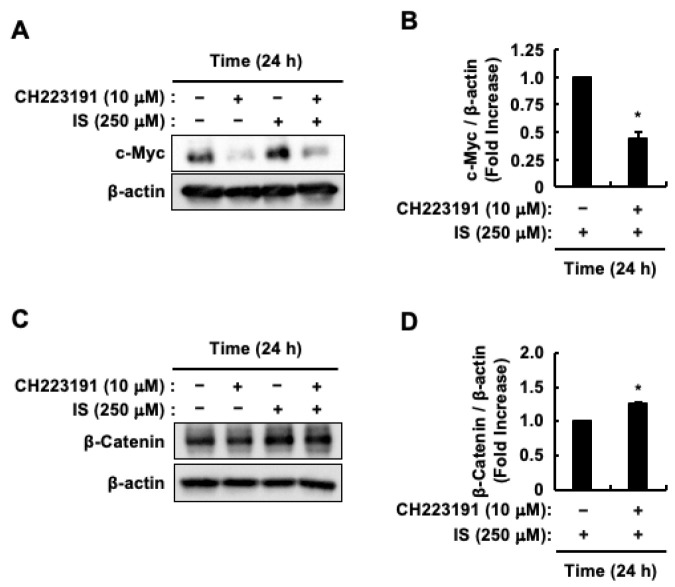
Impact of AhR on c-Myc and β-Catenin protein levels triggered by indoxyl sulfate in HCT-116 CRC cells. HCT-116 CRC cells were incubated with (+) or without (−) control (DMSO), indoxyl sulfate, or CH223191. (**A**) The effects of CH223191 on indoxyl sulfate-induced c-Myc and β-actin protein levels. (**B**) The band intensity of c-Myc in response to indoxyl sulfate alone and in combination with CH223191. (**C**) The effects of CH223191 on indoxyl sulfate-induced β-Catenin and β-actin protein levels. (**D**) The band intensity of β-Catenin in response to indoxyl sulfate alone and in combination with CH223191. The band intensities of c-Myc and β-Catenin were normalized to that of β-actin. The results are presented as mean values ± SE from three independent experiments for (**B**,**D**). * *p* < 0.05, vs. indoxyl sulfate alone. IS, indoxyl sulfate.

**Figure 5:**
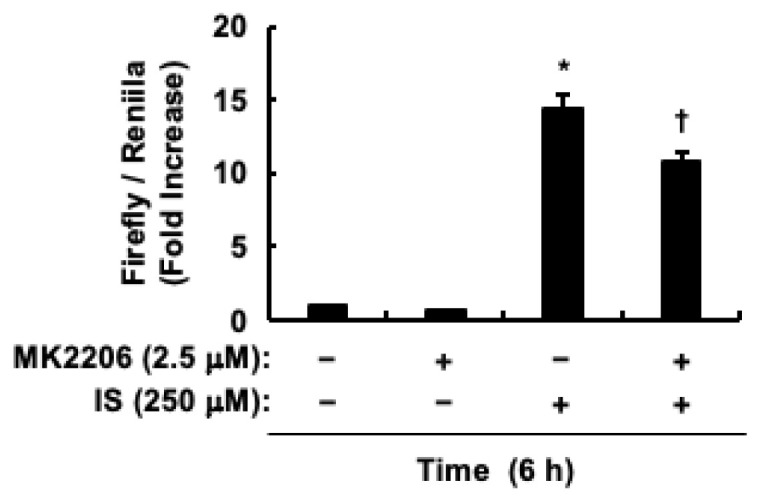
Impact of Akt on AhR transactivation triggered by indoxyl sulfate in HCT-116 CRC cells. The effects of MK2206 on indoxyl sulfate-induced increases in AhR transcriptional activity. HCT-116 CRC cells were incubated with (+) or without (−) control (DMSO), indoxyl sulfate, or MK2206. The results are presented as mean values ± SE of duplicates from three independent experiments. * *p* < 0.05, vs. the control solution (DMSO). ^†^ *p* < 0.05, vs. indoxyl sulfate alone. IS, indoxyl sulfate.

Considering the role of indoxyl sulfate in promoting HCT-116 CRC cell proliferation through AhR activation [48], we investigated the effect of CH223191, an AhR antagonist, on β-Catenin and c-Myc protein levels. CH223191 inhibited the indoxyl sulfate-induced increase in c-Myc protein levels (Figure 4A,B). Conversely, CH223191 promoted the increase in β-Catenin protein levels induced by indoxyl sulfate (Figure 4C,D). Since AhR activation, as well as Akt activation, was involved in the increase in c-Myc protein levels caused by indoxyl sulfate, we examined whether Akt activation affected AhR transcriptional activity. Since the increase in the protein levels of CYP1A1, a representative target gene of AhR, peaked 6 h after indoxyl sulfate stimulation [48], we also measured the transcriptional activity of AhR at the same stimulation time after pretreatment of HCT-116 CRC cells with MK2206. MK2206 partially suppressed indoxyl sulfate-induced AhR transcriptional activity (Figure 5). These findings suggest that indoxyl sulfate functions as a ligand for AhR, and that Akt activation induced by indoxyl sulfate leads to increased c-Myc protein levels by enhancing AhR transcriptional activity in HCT-116 CRC cells.

### 2.5. Enhanced Sensitivity of HCT-116 CRC Cells to EGF Was Caused by an Increase in EGFR Protein Levels Through Indoxyl Sulfate-Induced Activation of the AhR/c-Myc and Akt/β-Catenin/c-Myc Pathways

**Figure 6:**
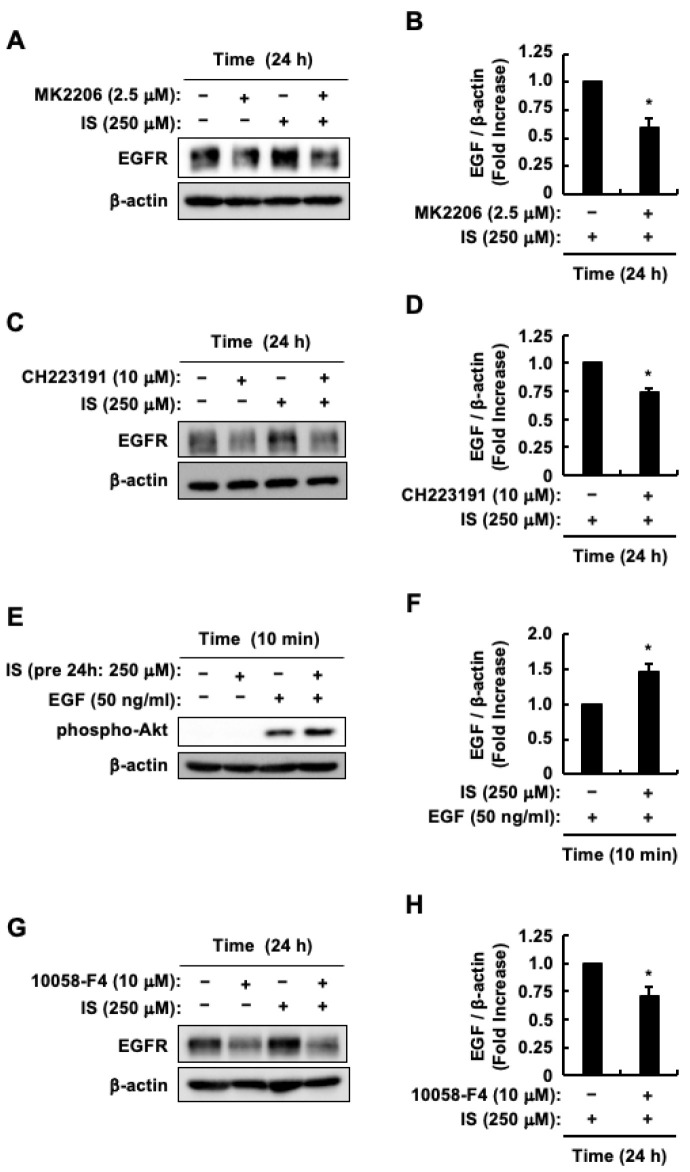
Impact of indoxyl sulfate on EGFR expression and enhanced EGF sensitivity in HCT-116 CRC cells. HCT-116 CRC cells were incubated with (+) or without (−) control (DMSO), indoxyl sulfate, EGF, MK2206 or CH223191. (**A**) The effects of MK2206 on indoxyl sulfate-induced EGFR and β-actin protein levels. (**B**) The band intensity of EGFR in response to indoxyl sulfate alone and in combination with MK2206. (**C**) The effects of CH223191 on indoxyl sulfate-induced EGFR and β-actin protein levels. (**D**) The band intensity of EGFR in response to indoxyl sulfate alone and in combination with CH223191. (**E**) The effects of indoxyl sulfate on EGF-induced Akt phosphorylation and β-actin protein levels. (**F**) The band intensity of phospho-Akt in response to EGF alone and EGF after pretreatment with indoxyl sulfate. (**G**) The effects of 10058-F4 on indoxyl sulfate-induced EGFR and β-actin protein levels. (**H**) The band intensity of EGFR in response to indoxyl sulfate alone and in combination with 10058-F4. The band intensities of EGFR and phospho-Akt were normalized to that of β-actin. The results are presented as mean values ± SE from three independent experiments for (**B**,**D**,**F**,**H**). * *p* < 0.05, vs. indoxyl sulfate alone. IS, indoxyl sulfate; EGF, epidermal growth factor; EGFR, epidermal growth factor receptor.

Indoxyl sulfate triggers the activation of both the AhR and Akt pathways, resulting in elevated levels of EGFR protein. This increase in EGFR protein levels enhances the sensitivity of HCT-116 CRC cells to EGF [48]. In addition, indoxyl sulfate induced an increase in c-Myc protein levels by activating AhR. It also upregulated β-Catenin protein levels through Akt activation (Figure 3A–H and Figure 4A,B). Additionally, c-Myc binds directly to the EGFR gene’s promoter region [55]. With these considerations in mind, we examined the potential involvement of c-Myc in the indoxyl sulfate-induced increase in EGFR protein levels. Our previous study demonstrated that the increase in EGFR protein levels peaked after 24 h of indoxyl sulfate stimulation [48]; therefore, we stimulated HCT-116 CRC cells with indoxyl sulfate for the same duration after treatment with various inhibitors. Consistent with the results of our previous study [48], the indoxyl sulfate-induced increase in EGFR protein levels was suppressed by CH223191 and MK2206 (Figure 6A–D), indicating that AhR and Akt activation is involved in the indoxyl sulfate-induced increase in EGFR protein levels. Furthermore, consistent with our earlier work [48], Akt activation by EGF was enhanced following a 24-h incubation with indoxyl sulfate. This enhancement occurred even without the presence of indoxyl sulfate during EGF stimulation (Figure 6E,F). This outcome suggests that indoxyl sulfate boosts EGF sensitivity in HCT-116 CRC cells through an increase in EGFR expression, driven by the activation of the AhR and Akt pathways. Based on these findings, we tested whether the increase in c-Myc protein levels induced by indoxyl sulfate led to an increase in EGFR protein levels. The c-Myc inhibitor 10058-F4 suppressed the indoxyl sulfate-induced increase in EGFR protein levels (Figure 6G,H). Collectively, these findings indicate that two distinct pathways regulate the increase in EGFR protein levels: namely, the AhR/c-Myc and Akt/β-Catenin/c-Myc pathways. This process, driven by indoxyl sulfate, leads to an enhanced sensitivity of HCT-116 CRC cells to EGF.

## 3. Discussion

**Figure 7:**
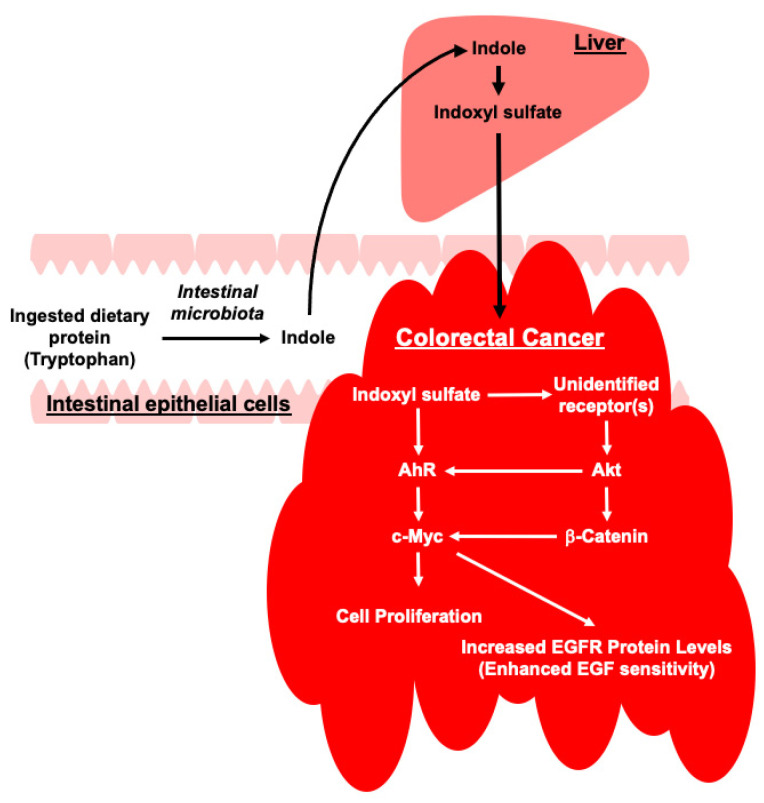
Schematic showing potential regulatory pathways by which indoxyl sulfate may exacerbate colorectal cancer (CRC) as chronic kidney disease (CKD) progresses. Indoxyl sulfate, a uremic toxin that accumulates in the blood of patients with CKD, induces proliferation of HCT-116 CRC cells and enhances their sensitivity to epidermal growth factor (EGF). This occurs through the activation of two distinct signaling pathways: the Akt/β-Catenin/c-Myc pathway and the AhR/c-Myc pathway. Activation of these pathways leads to the increased expression of c-Myc, a key oncogene involved in CRC development and progression. Furthermore, indoxyl sulfate increased EGFR protein levels via c-Myc-dependent mechanisms, thereby enhancing the responsiveness of CRC cells to EGF. These findings suggest that the accumulation of indoxyl sulfate in patients with CKD may contribute to the exacerbation of CRC by inducing CRC cell proliferation and augmenting EGF signaling. Targeting indoxyl sulfate may represent a potential therapeutic strategy for managing CRC in patients with concomitant CKD. AhR, aryl hydrocarbon receptor; EGF, epidermal growth factor; EGFR, epidermal growth factor receptor.

An increase in blood indoxyl sulfate concentration associated with CKD progression may also exacerbate CRC. Indoxyl sulfate appeared to stimulate CRC cell proliferation and increase responsiveness to EGF by activating two distinct signaling pathways: the Akt/β-Catenin/c-Myc and AhR/c-Myc pathways (Figure 7). The observation of the dual activation mechanisms suggests that indoxyl sulfate significantly exacerbates CRC. Therefore, further research is required beyond the cellular model presented shown in Figure 7 to fully elucidate the effect of indoxyl sulfate on the exacerbation of CRC with concomitant CKD. Animal studies and human clinical trials are required to validate and extend the knowledge gained from in vitro experiments. Such investigations could provide a thorough understanding of the mechanisms involved and potentially lead to targeted therapeutic interventions. These interventions could address both the underlying renal dysfunction in patients with CKD and its subsequent impact on cancer progression, providing a novel approach for the treatment of CRC in individuals with coexisting kidney disease. By developing strategies that combine cellular models with clinical applications, we can potentially improve the outcomes for patients with both CKD and CRC.

Anatomically, the colon is divided into right and left segments according to their embryological origin. Consequently, CRC may also be classified as a right-sided or left-sided malignancy. A defining characteristic of right-sided CRC is its higher frequency of mutations in the Kirsten rat sarcoma viral oncogene homolog (K-Ras) gene compared to left-sided CRC [56]. Furthermore, microsatellite instability (MSI) occurs at a significantly higher rate in right-sided CRC than in left-sided CRC [57,58]. MSI is characterized by DNA mismatch repair defects, primarily due to dysfunction of MutL homolog 1 (MLH1), the most prevalent gene associated with DNA mismatch repair. This dysfunction results in the accumulation of mutations within growth-regulatory genes, which subsequently promotes CRC progression [59]. HCT-116 CRC cells used in the present study were MLH1-deficient and harbored a K-Ras mutation, serving as a model for right-sided CRC. It is hypothesized that indoxyl sulfate has a more pronounced effect on right-sided CRC than on left-sided CRC, potentially serving as a biomarker [60]. Therefore, HCT-116 CRC cells could be an appropriate model for studying CKD-associated CRC, especially for analyzing the effects and mechanisms of action of indoxyl sulfate on these cells.

Our recent research indicates that indoxyl sulfate promotes HCT-116 CRC cell proliferation [48]. In this context, the systemic effects of CKD, including the accumulation of uremic toxins such as indoxyl sulfate, which may contribute to tumorigenic processes, have been discussed [47]. This is supported by our findings. However, one study suggests that indoxyl sulfate inhibits the growth and survival of CRC cells [61]. This apparent contradiction may be due to the different concentrations of indoxyl sulfate used. It is plausible that indoxyl sulfate concentrations comparable to serum levels in CKD promote the proliferation of CRC cells, whereas treatment of CRC cells with substantially higher concentrations of indoxyl sulfate may result in decreased proliferation and survival of CRC cells.

The present study showed that CH223191 pretreatment promoted an increase in β-Catenin protein levels induced by indoxyl sulfate. Therefore, AhR activation by indoxyl sulfate may lead to β-Catenin degradation. This speculation is supported by a previous report [62]. Conversely, some studies have reported that AhR activation does not lead to the degradation of β-Catenin [45,63,64]. These differences could stem from several factors such as variations in the expression of molecules in cells grown under diverse conditions. Therefore, the direct effect of AhR on β-Catenin remains controversial. Another possibility is that pretreatment with CH223191 enhances Akt activation caused by indoxyl sulfate, leading to higher β-Catenin protein levels [48]. Since the present study showed that Akt activation induced by indoxyl sulfate increases β-Catenin protein levels, the proposed mechanism appears to be plausible.

Although CH223191 pretreatment promoted an increase in β-Catenin protein levels in response to indoxyl sulfate, it simultaneously reduced c-Myc protein levels under comparable conditions. These results appear contradictory, because pretreatment with XAV-939 inhibited the increase in c-Myc protein levels induced by indoxyl sulfate. To explain this discrepancy, we considered the involvement of Scinderin (SCIN), whose expression is induced by AhR activation and has been reported to promote the accumulation of β-Catenin nuclei [65]. This finding implies that CH223191 pretreatment may suppress indoxyl sulfate-induced SCIN expression. Consequently, c-Myc protein levels did not increase, despite the increase in β-Catenin protein levels caused by indoxyl sulfate. Future research should investigate whether indoxyl sulfate-induced AhR activation causes an increase in SCIN expression and subsequently leads to the nuclear accumulation of β-Catenin protein, aspects that are beyond the scope of the present study.

Our present research demonstrated that indoxyl sulfate activates the Akt/β-Catenin/c-Myc and AhR/c-Myc pathways. This activation results in the upregulation of EGFR, thereby increasing the sensitivity of HCT-116 CRC cells to EGF. These findings suggest that indoxyl sulfate plays a role in the enhancement of EGF signaling in patients with CRC. EGFR is expressed in 25–75% of CRC cases [66], and its ligands, EGF and TGF-α, are found at higher levels in the mucosa surrounding malignant tumors than in the normal mucosa [66]. Moreover, EGFR expression is associated with poor prognosis and an increased risk of metastasis in CRC [66]. In addition to these findings, considering reports that AhR, β-Catenin, and c-Myc are involved in promoting metastasis [25,32,55], and that β-Catenin and c-Myc are involved in poor prognosis [20,67], CRC in patients with CKD, in whom the Akt/β-Catenin/c-Myc and AhR/c-Myc pathways are assumed to be activated by indoxyl sulfate, is expected to show high metastatic potential and poor prognosis.

AhR activation promotes resistance to chemotherapy in prostate and breast cancers, and choriocarcinoma [27,33,34]. Specifically, activation of the AhR/c-Myc pathway is considered to confer resistance to chemotherapy in prostate cancer [27]. Moreover, patients with RAS-BRAF wild-type mCRC and higher c-Myc expression levels show significantly faster and stronger resistance to anti-EGFR chemotherapy than those with lower c-Myc levels [20]. The current study revealed that the increase in c-Myc protein levels induced by indoxyl sulfate was associated with increased EGFR protein levels. Due to their reduced glomerular filtration rate, patients with CKD need to undergo chemotherapy at lower doses [68]. Therefore, chemotherapy may be largely ineffective against CRC in patients with CKD who have accumulated indoxyl sulfate in their blood.

The present and our previous studies demonstrated a role for AhR in causing the proliferation of CRC cells and metastasis to the liver [31,32,48], whereas another investigation suggested that AhR also possesses the capacity to inhibit the proliferation of CRC cells [28,29,30]. This apparent contradiction may arise from variations in the AhR ligands. For instance, in CKD, indoxyl sulfate contributes to disease progression through AhR [69], whereas statins suppress it by promoting the extracellular elimination of uremic substances via AhR activation [70]. Thus, targeting AhR may not effectively treat CRC in patients with CKD. Additionally, the present study suggested c-Myc as a promising therapeutic target for the treatment of CRC in patients with CKD. However, attempts to target c-Myc in clinical trials have not achieved the anticipated success due to various challenges [16]. The importance of c-Myc in maintaining tissue equilibrium and the existence of partially overlapping transcription factors (c-Myc, N-Myc, and L-Myc) that would need to be collectively targeted for effective treatments are among these explanations [16,71]. Based on these findings, AST-120 may be useful in delaying the progression of CRC associated with CKD. AST-120 reduces serum and urine levels of indoxyl sulfate; it absorbs and excretes indole, which is produced by intestinal bacteria in the colon of patients with CKD, through feces [39]. AST-120, which has been approved in Japan and Asia for delaying hemodialysis in patients with advanced CKD, has demonstrated a favorable safety profile [72]. Therefore, investigating AST-120’s effectiveness in slowing CRC progression in patients with CKD is valuable. On the other hand, hemodialysis is unlikely to effectively reduce CRC exacerbation in patients with CKD. This is because more than 90% of indoxyl sulfate binds to serum albumin [39], whereas conventional hemodialysis eliminates only approximately 31.8% of it from the blood [73]. Considering these findings and the average serum indoxyl sulfate level of approximately 250 μM in patients with ESRD [40], the post-hemodialysis level is 170.5 μM. This concentration exceeded the concentration used in the present study, as proliferation of HCT-116 CRC cells was observed even at 62.5 μM indoxyl sulfate in the present study.

Patients with CKD exhibit impaired absorption in the intestinal tract [74], resulting in an increased quantity of dietary protein components that are not absorbed in the small intestine, which subsequently reaches the large intestine. Furthermore, in vivo experiments have demonstrated that CKD decelerates intestinal motility [75], leading to the accumulation of dietary proteins. Consequently, the intestines of patients with CKD are more susceptible to the production of substantial amounts of indole, a precursor of indoxyl sulfate. Since *F. nucleatum*, a bacterium associated with the development of CRC, produces indole, its interaction with indoxyl sulfate may exacerbate CRC in patients with CKD.

## 4. Conclusions

The primary finding of our study was the link between increased blood levels of indoxyl sulfate and the advancement of CKD, which may also play a role in the worsening of CRC. This is important given the current lack of clarity regarding the molecular mechanisms underlying the role of CKD in worsening CRC. Specifically, our study indicated that indoxyl sulfate stimulates HCT-116 CRC cell proliferation and increases EGF sensitivity. This effect is mediated through the Akt/β-Catenin/c-Myc and AhR/c-Myc pathways, resulting in increased levels of EGFR protein. Furthermore, our earlier research demonstrated that AhR is not involved in Akt activation [48]. This suggests that a specific, but yet to be identified, receptor is responsible for activating the Akt/β-Catenin/c-Myc pathway. However, in the present study, we were unable to identify the novel receptors that activate this pathway. Future studies should aim to elucidate these mechanisms. In addition, by reducing *F. nucleatum* levels in the gut to improve intestinal health, we may be able to reduce elevated blood indoxyl sulfate levels, potentially reducing the severity of CRC. Future research should explore strategies to improve the gut environment, particularly through the use of prebiotics and probiotics.

## 5. Materials and Methods

### 5.1. Materials

Various reagents and antibodies were obtained from suppliers for the present study. Santa Cruz Biotechnology, Inc. (Dallas, TX, USA) was used to generate anti-β-actin antibody (C4). Anti-β-Catenin, anti-EGFR, anti-phospho-Akt, anti-phospho-GSK3β, and anti-c-Myc antibodies were supplied by Cell Signaling Technology, Inc. (Danvers, MA, USA). Peroxidase AffiniPure Goat Anti-Rabbit IgG (H+L) and anti-mouse IgG (H+L) antibodies were acquired from Jackson ImmunoResearch Laboratories, Inc. (West Grove, PA, USA). Nacalai Tesque Inc. (Kyoto, Japan) supplied Protease Inhibitor Cocktail (EDTA-free) (100×) and Phosphatase Inhibitor Cocktail. Biosynth (Staad, Switzerland) was used as the source of 3-Indoxyl sulfate, potassium salt. Penicillin–streptomycin solution (×100), dimethyl sulfoxide (DMSO), and Dulbecco’s modified Eagle’s medium (DMEM) with low glucose were obtained from Wako Pure Chemical Industries, Ltd. (Osaka, Japan). Cayman Chemical (Ann Arbor, MI, USA) provided CH223191 (AhR antagonist). Selleck Chemicals (Houston, TX, USA) supplied 10058-F4 (c-Myc inhibitor), MK2206 (Akt inhibitor), and XAV-939 (β-Catenin inhibitor). Fetal bovine serum (FBS) was procured from Biowest SAS (Nuaillé, France).

### 5.2. Cell Culture

HCT-116 CRC cells were obtained from RIKEN Cell Bank (Tsukuba, Japan). Cell cultivation was performed according to previously established protocols [48]. Cells were incubated in serum-free DMEM for 24 h before the experiments were commenced. DMSO was used as both the control and solvent for several compounds, including CH223191, 10058-F4, MK2206, and XAV-939. The final concentration of DMSO in the experimental setup was 0.1% (*v*/*v*).

### 5.3. Quantitation of Cell Proliferation

HCT-116 CRC cells underwent a 24-h exposure to either control solution (0.1% DMSO) or 10058-F4 (10 μM). Following this, the cells were treated with various concentrations of indoxyl sulfate or left untreated for 48 h. Cell proliferation was evaluated using the Cell Counting Kit-8 (Dojindo, Kumamoto, Japan), according to a previously established protocol [48].

### 5.4. Immunoblotting

HCT-116 CRC cells were exposed to control solution (0.1% DMSO), CH223191 (10 μM), or MK2206 (2.5 μM) for 30 min, or control solution (0.1% DMSO), 10058-F4 (10 μM), or XAV-939 (10 μM) for 24 h. The cells were then subjected to indoxyl sulfate (250 μM) treatment for different durations. Cell lysis was performed using a buffer as described in previous studies [42,45]. Proteins were separated using sodium dodecyl sulfate-polyacrylamide gel electrophoresis and transferred onto Immobilon-P polyvinylidene fluoride membranes (Millipore Inc., Bedford, MA, USA). The membranes were then incubated with specific antibodies: anti-c-Myc (1:1000), anti-phospho-Akt (1:1000), anti-phospho-GSK3β (1:1000), anti-β-Catenin (1:1000), anti-EGFR (1:1000), and β-actin (C4) (1:5000). Target protein detection was achieved using the Chemi-Lumi One L or Chemi-Lumi One Super system (Nacalai Tesque Inc., Kyoto, Japan) and subsequent analysis was performed using an ImageQuant LAS 4010 densitometer (GE Healthcare Life Sciences, Uppsala, Sweden). Quantification of the blotted proteins was conducted using ImageJ 1.53J software with the Band/Peak Quantification macro [76]. Protein levels were standardized to β-actin and presented as fold increases compared to the control.

### 5.5. Transfection and Luciferase Assays

To assess the transcriptional activity of AhR in HCT-116 CRC cells, pGL4.43[luc2P/XRE/Hygro] and pRL-SV40 plasmids (both from Promega, Madison, WI, USA) were transfected into cells utilizing FuGENE HD (Roche, Mannheim, Germany). After a 24-h incubation, the cells were subjected to either a control solution (0.1% DMSO) or MK2206 (2.5 μM) for 30 min. Subsequently, cells were exposed to indoxyl sulfate (250 μM) or left untreated for 6 h. Measurements were then conducted using a Junior LB9509 luminometer (Berthold Technologies, Bad Wildbad, Germany) following previously established protocols [44,46].

### 5.6. Statistical Analysis

The results are presented as mean values with SE. For statistical analysis, Dunnett’s test was used for Figure 1, while the Tukey–Kramer test was used for Figure 2C and Figure 5. The Student’s *t*-test was applied to Figure 2B, Figure 3B,D,F,H, Figure 4B,D and Figure 6B,D,F,H. Statistical evaluations were performed using Microsoft Excel 2011 (Microsoft Corp., Redmond, WA, USA) and Statcel 4 software (OMS Publishing Co., Saitama, Japan). Statistical significance was set at *p* < 0.05.

## Data Availability

The original contributions presented in this study are included in this article. Further inquiries can be directed to the corresponding author.
